# A parent-led intervention to promote recovery following pediatric injury: study protocol for a randomized controlled trial

**DOI:** 10.1186/s13063-019-3207-9

**Published:** 2019-02-18

**Authors:** Meghan L. Marsac, Ginny Sprang, Leila Guller, Kristen L. Kohser, John M. Draus, Nancy Kassam-Adams

**Affiliations:** 1 Department of Pediatrics, Kentucky Children’s Hospital, Lexington, KY USA; 2 College of Medicine, Kentucky Children’s Hospital, University of Kentucky, 800 Rose St, MN 472, Lexington, KY 40536 USA; 3 Center on Trauma and Children, University of Kentucky, Lexington, KY USA; 4 College of Arts and Sciences, University of Kentucky, Lexington, KY USA; 5 Center for Injury Research and Prevention, Children’s Hospital of Philadelphia, Philadelphia, PA USA; 6 Perelman School of Medicine, University of Pennsylvania, Philadelphia, PA USA; 7 Department of Surgery, University of Kentucky, Lexington, KY USA

**Keywords:** Cellie Coping Kit, Early intervention, Emotional health, Coping, Appraisals, Child injury, Parent intervention

## Abstract

**Background:**

Injury is one of the most prevalent potentially emotionally traumatic events that children experience and can lead to persistent impaired physical and emotional health. There is a need for interventions that promote full physical and emotional recovery and that can be easily accessed by all injured children. Based on research evidence regarding post-injury recovery, we created the Cellie Coping Kit for Children with Injury intervention to target key mechanisms of action and refined the intervention based on feedback from children, families, and experts in the field. The Cellie Coping Kit intervention is parent-guided and includes a toy (for engagement), coping cards for children, and a book for parents with evidence-based strategies to promote injury recovery. This pilot research trial aims to provide an initial evaluation of the impact of the Cellie Coping Kit for Children with Injury on proximal targets (coping, appraisals) and later child health outcomes (physical recovery, emotional health, health-related quality of life).

**Method / Design:**

Eighty children (aged 8–12 years) and their parents will complete a baseline assessment (T1) and then will be randomly assigned to an immediate intervention group or waitlist group. The Cellie Coping Kit for Injury Intervention will be introduced to the immediate intervention group after the T1 assessment and to the waitlist group following the T3 assessment. Follow-up assessments of physical and emotional health will be completed at 6 weeks (T2), 12 weeks (T3), and 18 weeks (T4).

**Discussion:**

This will be one of the first randomized controlled trials to examine an intervention tool intended to promote full recovery after pediatric injury and be primarily implemented by children and parents. Results will provide data on the feasibility of the implementation of the Cellie Coping Intervention for Injury as well as estimations of efficacy. Potential strengths and limitations of this design are discussed.

**Trial registration:**

Clinicaltrials.gov, NCT03153696. Registered on 15 May 2017.

**Electronic supplementary material:**

The online version of this article (10.1186/s13063-019-3207-9) contains supplementary material, which is available to authorized users.

Injury is among the most common potentially traumatic experiences for children. In the US alone, 20 million children suffer unintentional injuries annually [1–3]. Injuries can result in impairments to long-term physical and/or emotional health. For example, 18% of children presenting to the Emergency Department (ED) for injury report functional impairment five months later [4] and 13–19% (i.e. 3.8 million/year in the US) experience impaired emotional health (e.g. post-traumatic stress [PTSS] or depression) [5, 6]. Rural youth are at even higher risk for impairment as they are more likely to have inadequate health insurance [7] and live in areas with insufficient healthcare services [8]. While it is essential to promote full physical and emotional recovery, there are challenges to developing interventions that are effective, developmentally appropriate, and practical for wide reach. The strong evidence base on post-injury recovery has not yet led to effective, accessible interventions for all injured children [9, 10].

Physical and emotional health impairments in children after an injury are often unrecognized; if they are recognized, families frequently do not know how or where to obtain assistance [11]. Current resources tend to focus on illness (rather than injury) and often require the assistance of professionals who have mental health training [12, 13]. Only three interventions show promise for children coping with the everyday challenges of injury recovery: Kids and Accidents (reduced anxiety); [14] Coping Coach (reduced PTSS) [15]; and The Child and Family Traumatic Stress Intervention (reduced PTSS) [16]. However, none of these resources directly involve caregivers as coaches in a flexible, self-guided, and low-cost intervention.

To fill this gap, we created the Cellie Coping Kit for Children with Injury intervention based on the evidence regarding mechanisms of action in psychological and physical recovery after injury. The Coping Kit targets specific mechanisms by promoting adherence to medical regimens, positive cognitive appraisals, and adaptive coping behaviors [17–24]. The development process for the Cellie Coping Kit included substantial feedback from children, families, and experts in the field (e.g. child life specialists, nurses, pediatricians, physical therapists, psychologists, social workers, trauma surgeons). The Cellie Coping Kit for Children with Injury is developmentally appropriate for children aged 8–12 years and allows children and caregivers to tailor strategies to their unique injury experience, enabling families to identify their most important stressors and the coping strategies that work best for them. By involving caregivers as coaches in this intervention, we draw on evidence supporting a model of parental socialization of coping to strengthen the child’s existing support system. See Fig. 1 for sample coping card content. A particular strength of the Cellie intervention is that it is portable, allowing children and their caregivers to use the Kit across settings (e.g. at home, at the hospital, during procedures, at follow-up appointments) and with providers (e.g. doctors, nurses, physical/occupational therapists) ensuring that the intervention is available at the time an injury-related stressor arises. See Marsac et al. for a detailed description of intervention development [25].

**Fig. 1:**
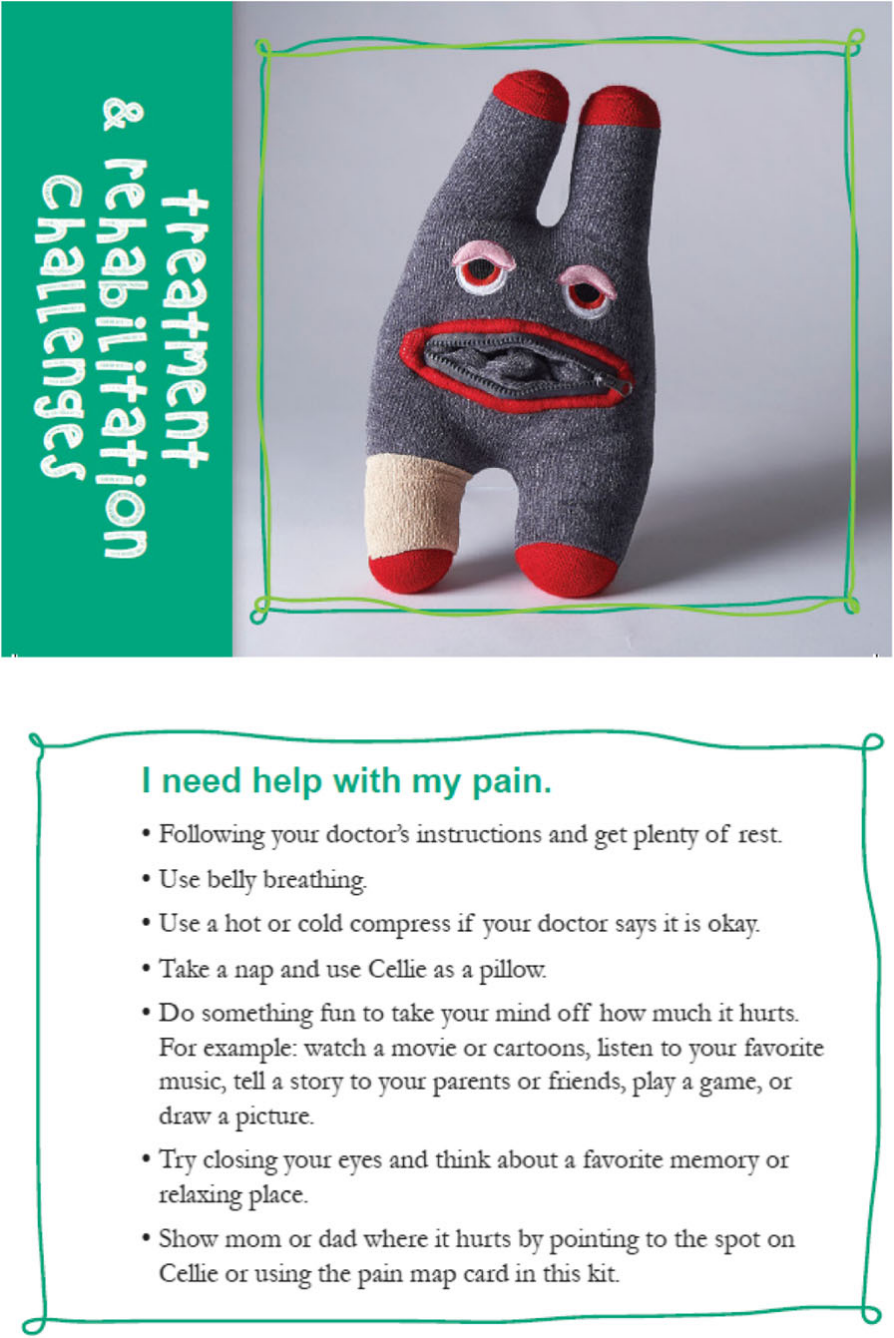
Sample coping card from the Cellie Coping Kit for Children with Injury

## Mechanisms of action

Two conceptual models have informed the Cellie Intervention’s targeted mechanisms of action: the Health Belief Model (HBM) as applied to adherence [26, 27]; and the cognitive model of appraisals and coping in response to potentially traumatic events such as injury [28–31]. Empirical evidence garnered over the last 20 years supports the HBM as an explanatory model for adherence in a range of pediatric populations [32–34]. This model suggests that health behaviors are influenced by individual perceptions of threat and benefits of health and health behaviors [26, 27]. Empirically supported cognitive models of response to trauma exposure posit a central role for coping strategies in emotional recovery. Maladaptive appraisals of the potentially traumatic event (i.e. unrealistic threat appraisals of injury and treatment) can lead to behavioral strategies (i.e. maladaptive coping such as avoidance) that either directly result in psychological symptoms and/or prevent development of adaptive appraisals [35, 36]. There is strong empirical support for the role of cognitive coping in children’s post-injury emotional health: children’s perception of threat [17–20] and negative appraisals about vulnerability to future harm [37, 38] are related to worse emotional health. Integrating the HBM and cognitive models can help to explain barriers to adherence and pathways to health outcomes. Children who engage in more avoidant coping strategies based on threat perceptions (appraisals) demonstrate lower adherence, [39] resulting in worse physical health [23, 24]. Greater avoidant coping has been associated with poorer health-related quality of life (HRQOL) in children with asthma [40] and sickle cell disease, [41] while greater social support seeking has been linked to better HRQOL in children with arthritis [42]. Thus, research strongly implicates adherence and coping during early recovery as mechanisms of action for children’s emotional and physical health.

## Current study

In this paper, we describe the design of a pilot randomized controlled trial (RCT) which aims to evaluate the impact of the Cellie Coping Kit for Children with Injury on proximal targets (adherence, coping behaviors) and later child health outcomes (physical recovery, emotional health, HRQOL). There are three primary objectives of the pilot RCT: (1) to assess the feasibility of the intervention; (2) to assess the initial efficacy of the intervention in changing hypothesized mechanisms of action; and (3) to assess preliminary efficacy of the intervention in changing hypothesized child health outcomes. In this paper, we describe the design of this pilot RCT, including strengths and limitations.

## Cellie Coping Kit for Children with Injury Intervention Description

The Cellie Intervention is led by parents (with support from interventionists). The intervention’s portable, engaging design and active partnership with parents as consistently available coaches allows families to use it anywhere (at home, during procedures) ensuring the child is supported at the time the injury-related stressor arises. The intervention is tailored for middle childhood: the skills promoted (adherence, coping) harness communication skills acquired during this period. The Cellie intervention includes: (1) a toy to promote engagement (playing with the toy is not an active ingredient); 2) caregiver book; and 3) coping cards. Skills are presented in a way usable by most families. Intervention use is tailored to each child’s injury experience. For example, if a child is in pain, a parent can find pain-specific strategies in the book and coping cards and help their child implement these strategies. The intervention can be used during medical care (e.g. when a child is getting an IV), follow-up care (e.g. during physical therapy), or at home (e.g. when in pain after surgery). See Fig. 1 for sample coping card content. The Cellie Intervention is universal, in that it can be implemented across the full spectrum of psychosocial functioning at the intensity of families’ choosing.

Pilot research supports the acceptability of the Cellie Intervention for injured children. In a study of children with injuries and their parents (*n* = 60 child–parent dyads), almost all children and parents who completed follow-up found the intervention helpful (e.g. 95% of parents would recommend it to others) and most reported learning new skills (e.g. how to facilitate a conversion about the injury) [25]. The trial described here will provide initial data on the intervention’s impact on proximal and longer-term health outcomes.

## Method

### Participants

Participants will include 80 children with injury and one parent per child. Participants will be drawn from a medical institution predominantly serving a rural population. Eligibility criteria for this study include: (1) injury severe enough to warrant emergency medical treatment or hospital admission; and (2) child aged 8–12 years. Exclusion criteria for this study include: (1) language barriers or cognitive limitations preventing comprehension of intervention materials or assessments; or (2) injury resulting from child abuse/family violence.

### Study design

Before approaching potential participants, randomization will be determined using a random-number generator. Sealed envelopes specifying study condition will be prepared by research personnel not otherwise involved in the study. Research assistants and participants will be blind to condition until baseline assessments are completed, at which time envelopes will be opened to reveal whether they will be in the intervention or waitlist condition. Medical personnel will not be explicitly told of participants’ study condition; however, they may observe the Cellie Coping Kit in the child’s treatment room, so full blinding is not possible. Additionally, because this will be a pilot study with limited staffing, it is not possible to blind those who are conducting follow-up assessments.

**Fig. 2:**
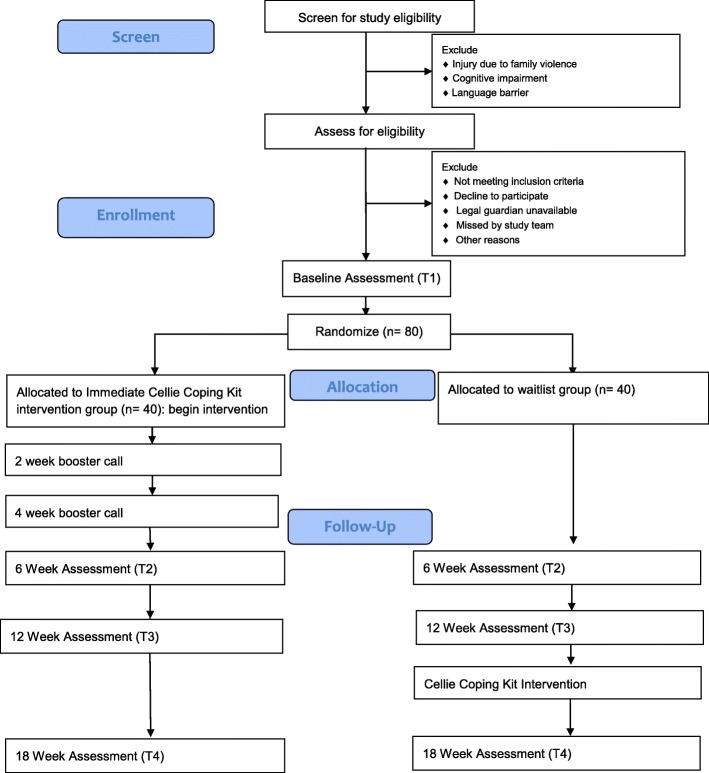
CONSORT diagram displaying planned study enrollment and randomization

**Fig. 3:**
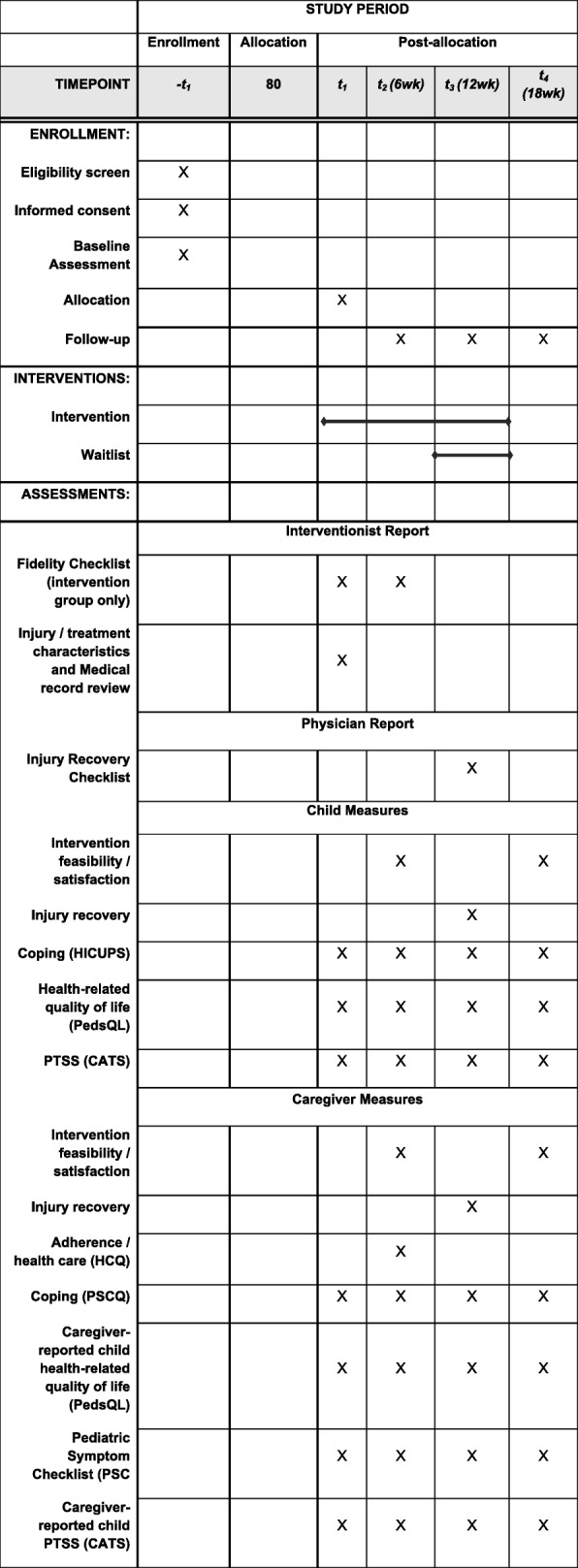
Schedule of enrollment, interventions, and assessments

Following caregiver consent and child assent, participants will complete a baseline assessment (T1) including measures of coping, HRQOL, and psychological symptoms. Families will then be randomized to the immediate Cellie Intervention (*n* = 40) or Waitlist control (n = 40). Those in the intervention condition will begin the intervention immediately: Cellie interventionists (trained research assistants [RAs]) will meet with families, explain the purpose of the intervention, and provide an overview of the intervention materials. Interventionists will then work with families to identify their three most pressing injury-related challenges, determine which information is most relevant, and help families role-play using these strategies. At two and four weeks after baseline, families in the intervention group will be offered a booster session via phone to discuss use of the Kit, problem-solve difficulties, and apply the Kit to new stressors. Families requiring more intensive support will be referred to other services, as this intervention is not a substitute for psychological treatment. Participants assigned to the waitlist group will receive standard care until they are given access to the Cellie Coping Kit intervention (via mail) after the T3 assessment. Standard care will include family-centered medical care, access to support from child life specialists, and referrals to a social worker, psychologist, and/or psychiatrist if the medical team has concerns about a child’s emotional health. At T3, participants will be provided written instructions on how to use the coping kit (rather than an in-person introduction) and contact information for questions related to the intervention. This group will not receive the booster sessions. Interventionists will complete fidelity measures at T1, booster sessions, and T2 (see Table 1). At 6 weeks (T2), 12 weeks (T3), and 18 weeks (T4) after baseline, participants will repeat T1 measures. In addition, parents will report on adherence at T2 (see Table 1). Children, parents, and a physician (blinded to participants’ condition) will also rate physical recovery at T3. Assessments will be completed via phone or Redcap [43]. It is estimated that participants will complete T1 assessments in approximately 25 min and follow-up assessments in about 20 min. To improve retention, families will be contacted five times each via phone for follow-up assessments. If research assistants are unable to contact families for follow-ups via phone, an electronic link to the follow-up survey on REDCap will be sent via email. If the family does not complete assessments via phone or Redcap [43], families will be asked to complete assessments via mail. The study protocol has been reviewed and approved by the Institutional Review Board at the University of Kentucky and is registered at clinicaltrials.gov. See Fig. 2 for CONSORT diagram [44] and Fig. 3 for the SPIRIT checklist and Addiitional file 1 for SPIRIT checklist.

**Table 1:** Constructs and measures for RCT

Construct/Measure	Assessment^a^			
Interventionist Report	T1	T2	T3	T4
Fidelity/Fidelity checklist	X (intervention group)	X (intervention group)		
Injury and treatment characteristics/Medical record review/abstraction	X			
Physician report	T1	T2	T3	T4
Physical Injury Recovery			X	
Child Self-Report	T1	T2	T3	T4
Intervention feasibility/Satisfaction Questionnaire [45, 46]		X (intervention group)		X (waitlist group)
Physical Injury Recovery			X	
Coping/How I Coped Under Pressure Scale (HICUPS) [47]	X	X	X	X
Health-related quality of life/Pediatric Quality of Life Inventory (PedsQL) [48]	X	X	X	X
Child and Adolescent Trauma Screen (CATS) [54]	X	X	X	X
Parent Self-Report	T1	T2	T3	T4
Intervention feasibility/Satisfaction Questionnaire [45, 46]		X (intervention group)		X (waitlist group)
Physical Injury Recovery			X	
Adherence/Health Care Questionnaire (HCQ)		X		
Coping Assistance/Parent Socialization of Coping Questionnaire (PSCQ) [47]	X	X	X	X
Health-related quality of life/PedsQL [49, 50]	X	X	X	X
Child Psych Symptoms/Pediatric Symptom Checklist (PSC) [51–53]	X	X	X	X
Child and Adolescent Trauma Screen- Caregiver Report (CATS) [54]	X	X	X	X

^a^Time of assessment: T1 = baseline; T2 = 6 weeks; T3 = 12 weeks; T4 = 18 weeks

### Procedure

Potential participants will be identified via the electronic health record. A RA will approach the caregivers of an eligible child in their hospital or clinic room, explain the study, and invite caregiver and child participation. Next, RAs will obtain informed consent (from parents/ guardians) and assent (from children). After completing the T1 assessment (Table 1), child participants will open a pre-prepared envelope (RAs will be blind to study condition before this) determining whether they will be randomized to the intervention or waitlist group.

### Assessments

#### Intervention use, satisfaction, and engagement

The Satisfaction Questionnaire [45, 46] was adapted from previous research and contains 22 items assessing fidelity, acceptability, usage, and barriers to using the intervention. This questionnaire was created for this study and is designed to gather overall impressions of and satisfaction with the Cellie Coping Kit intervention. It is divided into three sections: (1) several open-ended questions elicit strengths and areas for improvement; (2) items rated yes/no and on a 3-point Likert scale (yes, maybe, and no) ask the respondent to assess the intervention’s appeal, functionality, and the trustworthiness and comprehensibility of the intervention content; and (3) several open-ended questions assess how families engaged in the intervention at home and any barriers incurred to completing the intervention at home. Child and parent participants will both be asked to complete this questionnaire.

#### Adherence

The Adherence/Health Care Questionnaire (HCQ) will be used to assess adherence and contains eight categories of items specific to following discharge instructions (e.g. dietary and activity restrictions, follow-up clinical services, wound/drain/line care, medications).

#### Coping and assistance

The How I Coped Under Pressure Scale (HICUPS) [47] will assess the child’s use of adaptive coping strategies with regard to his or her injury and recovery. HICUPS has well-established reliability and validity. Due to the length of the scale, only specific subscales which correspond to content covered in the Cellie tool will be administered.

The Parental Socialization of Coping Questionnaire (PSCQ) [47] will assess parental (caregiver) coaching of child’s coping strategies specific to injury-related stressors. The measure parallels the HICUPS child measure described above, but parents are asked to rate the extent to which they have encouraged versus discouraged each specific child-coping strategy. Reliability and validity of the measure have been established. Due to the length of the scale, only specific subscales which correspond to content covered in the Cellie tool will be administered.

#### Health-related quality of life

The Pediatric Quality of Life Inventory (PedsQL) [48] is a well-validated measure of child HRQOL. It is developmentally appropriate, with child self-report and parent-report instruments available for ages 2–18 years. The PedsQL has four scales with a total of 23 items: Physical health / physical functioning (eight items); Psychosocial health / emotional functioning (five items); Psychosocial health / social functioning (five items); and Psychosocial health / school functioning (five items).

The PedsQL Caregiver Report [49, 50] consists of 23 items and parallels the child-report PedsQL above. This measure has well-established psychometric properties [49, 50].

#### Psychological symptoms

The Pediatric Symptom Checklist [51–53] is a 35-item validated questionnaire assessing parent-report of child emotional symptoms. The measure yields a total score and subscales for internalizing, conduct, and attention symptoms [51–53].

#### Post-traumatic stress symptoms

The Child and Adolescent Trauma Screen (CATS) [54] will assess post-traumatic stress disorder (PTSD) severity and PTSD diagnostic status. The CATS maps directly onto DSM-5’s criteria for PTSD: intrusions; avoidance; negative alterations in cognitions and mood; and hyperarousal. Traumatic events are elicited using a 15-item structured PTE checklist. PTSS are measured by 20 items rated on a 0–4 scale.

The CATS Caregiver Report [54] assesses child and adolescent PTSD severity and diagnostic status via caregiver report. The CATS caregiver report assesses the same areas as the child version described above, using the same structure.

#### Child physical injury recovery

Physicians (blinded to condition) will review the medical record and rate child recovery on a 3-point scale: worse than expected; as expected; or better than expected. Parents and children will also complete recovery measures, each ranking two injury-recovery items on a 3-point scale.

#### Fidelity

After introducing the Cellie Coping Kit to families, interventionists will complete a questionnaire to rate parents’ and children’s reactions to the content of the intervention. Following Mowbray et al. [55], interventionists will rate the extent of parents’ and children’s attentiveness, engagement, and receptiveness during the intervention training.

### Sample size

A goal of this pilot RCT is to estimate effect sizes for a full-scale RCT. Within the constraints of a pilot study, we aim to have reasonable power to detect a clinically meaningful effect for mechanisms of action (T2 adherence, coping) and child health outcomes (T3 physical recovery, HRQOL, emotional health symptoms). A sample of *n* = 80 (40 per condition; 68 [85%] retained to T3) can detect a 0.7 effect size between groups at T3 (ANCOVA) with 80% power and α = 0.05. Retention rate will be tracked and considered in designing subsequent research trials.

### Data management

All data will be collected on tablets and stored in the REDCap (Research Electronic Data Capture) database hosted at The University of Kentucky. REDCap is a secure, web-based application designed to support data capture for research studies, providing: (1) an intuitive interface for validated data entry; (2) audit trails for tracking data manipulation and export procedures; (3) automated export procedures for seamless data downloads to common statistical packages; and (4) procedures for importing data from external sources. To minimize risks of breach of confidentiality or invasion of privacy all data and records generated during this study will be kept confidential in accordance with Institutional policies and HIPAA on subject privacy. The Investigator and other site personnel will not use such data and records for any purpose other than conducting the study. Participants’ identities will be disguised by a unique identification number, which will appear on all questionnaire materials, instead of their name. The identification numbers will be linked with participant names only in a password-protected database and in a locked file with a master list of participants for case-management records and follow-up contacts. All case documents will be stored in a locked file. Consent documentation will be stored separately, in a locked file. All case documents and consent documents will be retained for seven years or until the study is completed, whichever is longer.

### Data analysis

For all analyses, we will include covariates of child age, sex, and injury characteristics (e.g. severity, length of recovery, type of procedures). Missing data will be imputed in cases in which at least 50% of a measure’s subscale is complete; in cases in which > 50% of the items on a given subscale are missing, participants’ will be dropped from analyses requiring those subscales but will be retained in the overall study. Regarding the primary objective, examining intervention feasibility (fidelity, acceptability, implementation, cost), we will perform descriptive statistics that will summarize: (1) fidelity, i.e. when, how, and what parts of intervention were implemented (child, parent, interventionist report), engagement (child, parent, interventionist report); (2) acceptability, i.e. child/parent satisfaction questionnaire and interview data; (3) implementation, i.e. selection of target stressors, strategies used, barriers identified (interview, session data), rate of booster session completion; and (4) cost, i.e. resources required (session time, additional family contact, booster calls, training/supervision, kit costs). We will consider the intervention feasible if: (i) > 50% of the participants in the intervention group report implementing the intervention; and (ii) > 75% of participants who implemented the intervention report high satisfaction with the intervention. Data on what parts of the intervention were used and the cost will inform future intervention development and research.

To determine the intervention’s efficacy and analyze mechanisms of action and health outcomes, our primary analytic approach will be analysis of covariance (ANCOVA) for initial estimation of effect sizes. ANCOVA can adjust for baseline group differences (intervention versus waitlist) if imbalances occur despite randomization. The dependent variable in each ANCOVA will be a follow-up measure (e.g. T2 adherence, coping; T3 HRQOL, psychological symptoms), with corresponding T1 score as the covariate. Thus, we will examine intervention effects at T2 and T3 while controlling for T1 differences (comparing the intervention group to the waitlist group). We will explore mechanisms of actions at T2 as possible mediators of the intervention effect on health outcomes at T3 via multivariable regression analysis. Data collected at T4 will be exploratory and will also be examined using ANCOVAs to allow us to examine potential intervention timing effects.

### Adverse event (AE) monitoring

Clinical AEs will be monitored throughout the study. Since the study procedures are not greater than minimal risk, serious AEs are not expected. If any unanticipated problems related to the research involving risks to individuals or others happen (including serious AEs), these will be reported to the institutional review board (IRB).

### Access to data

The principal investigators and co-investigators will have access to the final trial dataset. There are no limits to investigators by external agencies.

### Dissemination of data

Results of this study will be disseminated in full in professional journals, national, and international conferences. A lay summary of study results will be provided on ClinicalTrials.gov upon study completion. Plans for public access to data in an appropriate database will be developed.

### Protocol modifications

Although major protocol modifications are not anticipated at this point, any important modifications will be requested through a modification request form to the University of Kentucky’s Medical IRB and communicated to all study personnel.

### Authorship

Authorship will be granted to those who make notable contributions to the study.

## Discussion

Pediatric injury places children at significant risk for long-term impaired emotional and physical health [1, 2, 4, 5, 56]. While there is strong consensus that interventions to support injury recovery are essential and that better understanding intervention targets will increase effectiveness, interventions remain in their infancy and many have not been effective in promoting physical and emotional health [12, 57, 58]. Evidence suggests that adherence and coping may serve as key mechanisms of action for improving children’s post-injury health [18, 37, 59]. By partnering with parents [60, 61] and targeting children’s adherence and coping directly [18, 37, 59], the Cellie Coping Kit intervention is an ideal candidate for promoting full recovery in millions of children.

Building on promising findings from pilot studies [25], the current RCT goals represent an important next step in the advancement of the Cellie Coping Intervention for Injury. The strengths and potential positive outcomes of implementing a waitlist control RCT design with multiple informants are numerous. The current study design obtains data from both the child and parent perspective on physical recovery, emotional recovery, and response to the intervention. In addition, the design includes the physicians’ perspective on physical recovery and Cellie interventionists’ perspectives on intervention engagement / uptake. Combining data from multiple informants will provide rich information specific to the targets (i.e. adherence, coping behaviors, physical recovery, emotional health, HRQOL) and intervention engagement. Collecting data on cost, and on engagement from multiple perspectives (child, parent, and interventionist), will lay the ground work for more research on intervention dissemination and implementation. The longitudinal study design will allow us to examine change over time and investigate both short- and long-term effects of the intervention. Evaluating the level of support that a family needs to implement the Cellie Intervention is also a study strength. The role of the interventionist in this intervention has been designed so that advanced credentialing (i.e. licensure or certification) is not necessary. Rather, a wide range of health and behavioral healthcare workers can be trained to teach and support the use of the Cellie Coping Kit, facilitating implementation and sustainability of the approach. This is especially important in many rural healthcare professional shortage areas, where professionals with advanced degrees are in short supply, yet high demand. The design of this study will allow us to explore whether Cellie interventionists are necessary (Cellie intervention group) or whether the family can use the intervention with no additional instruction (waitlist group). The use of a waitlist control group will allow group comparisons at T2 and T3, with the additional possibility of examining intervention timing effects at T4. In addition, the waitlist design ensures that all participants will be offered the Cellie Coping Kit for Injury intervention addressing the overarching goal of the team to provide evidence-based resources to promote injury recovery. The target population of this study is also a strength. Much research on adherence/health to date has been conducted with chronic illness populations rather than injury [32–34]. Injury is in many ways qualitatively distinct from other health conditions in its treatment and long-term physical and emotional impact. In addition, this research protocol will include many families from rural communities, an underserved and under-researched population [62]. Including several strategies of collecting follow-up data (via phone, Internet, mail) will contribute to our understanding of how to engage and retain participants from rural communities at follow-up time points.

We recognize a number of potential limitations of this study design. Families may face some barriers to implementing the Cellie Coping Kit, such as difficulty understanding the materials or busy schedules interfering with the intervention. Thus, there may be variation in intervention usage within the intervention and waitlist control groups. We will address this by examining usage per child and parent report; however, the Kit’s portability and child- and parent-driven usage mean that it is not feasible to include behavioral observations to capture actual usage in the child’s environment. Other potential limitations include the possibility of lower than expected retention rates or logistical challenges (e.g. problems contacting participants by phone) that can arise in research with families living in rural communities. Any retention challenges will provide valuable information in planning for future research and intervention implementation. Other limitations of this RCT could include recruitment challenges and potential sampling bias. The research team will work to avoid such challenges by closely monitoring eligibility, recruitment, enrollment, and retention. Strategies will be adjusted as necessary to ensure recruitment goals are met and sample characteristics are reasonably balanced. Given the limited scope and funding for this pilot study, blinding will be limited; research staff and participants will be blind through the baseline assessment but not at subsequent follow-up assessments. To mitigate bias, research staff will be trained to follow a structured protocol to ensure all follow-up assessments are conducted in a similar manner. Because the medical team may directly observe intervention materials, it is not feasible to keep them blinded from study condition. Finally, this research design is a pilot study; thus, future research with a larger sample will be necessary to fully explore intervention effects.

In summary, the current waitlist control RCT aims to examine intervention feasibility and provide an initial estimate of impact. The data obtained in this study will support the larger program goal of using a translational research approach to develop an effective, low-cost mechanism for teaching adaptive coping strategies to children with injury and their families. If results suggest that the intervention helps promote physical and emotional health among children after experiencing an injury, then the Cellie Coping Intervention could be a low-cost mechanism to disseminate evidence-based strategies to support recovery from injuries in medical settings. The accessibility and user-driven design of the intervention makes it ideal for distribution across diverse settings and individuals. If shown effective in children with injury, there may also be avenues for broader application across other pediatric medical conditions.

## Trial status

This study is approved as protocol 17–0187-P1G at The University of Kentucky. Study enrollment began on 1 August 2017. It is estimated that recruitment will be completed by 1 June 2019, with a study completion date of 1 June 2020.

## Additional file

Additional file 1: SPIRIT checklist. (DOCX 25 kb)
